# Assessment of Respiratory Health Implications of Vaping: A Systematic Review of Toxicity Mechanisms and Adverse Effects of Electronic Nicotine Delivery Systems

**DOI:** 10.7759/cureus.69236

**Published:** 2024-09-11

**Authors:** Muhammad Mughis, Muhammad Ahmad, Hamayun Rashid, Anum Nasir, Hassan Mukarram, Sadia Chaudhary, Salman Tariq, Tahir Zaman

**Affiliations:** 1 Acute Medicine, University Hospitals Coventry & Warwickshire, Coventry, GBR; 2 Internal Medicine, Punjab Medical College, Faisalabad, PAK; 3 Emergency Medicine, University Hospitals Coventry & Warwickshire, Coventry, GBR; 4 Pediatric Emergency Medicine, Lady Reading Hospital, Peshawar, PAK; 5 Internal Medicine, Barking, Havering and Redbridge University Hospitals NHS Trust, London, GBR; 6 Behavioral Sciences, Rahbar Medical & Dental College Lahore, Lahore, PAK; 7 General Internal Medicine, East Lancashire Hospitals NHS Trust, Blackburn, GBR; 8 General Medicine, Lahore General Hospital, Lahore, PAK

**Keywords:** adverse effects, adverse mechanism, electronic nicotine delivery system, respiratory health implication, toxicity mechanism.

## Abstract

E-cigarettes are thought to aid in tobacco smoking cessation, but there are concerns about their overall effectiveness and safety for the general population, particularly adults. This review aims to investigate the mechanisms of toxicity and adverse effects of e-cigarettes on the respiratory system, comparing these effects with those of conventional smoking. A systematic review was conducted following the Preferred Reporting Items for Systematic Reviews and Meta-Analyses (PRISMA) guidelines. Searches were performed on PubMed, Embase, and the Cochrane Library using keywords, controlled vocabulary, and text words, with the following criteria: studies published in English from 2014 to 2024, open access, peer-reviewed, and full-text availability. Study selection, quality assessment, and data extraction were carried out by two independent reviewers. The Cochrane Risk of Bias 2.0 tool was used to evaluate the risk of bias in included randomized controlled trials, and the Grading of Recommendations Assessment, Development and Evaluation (GRADE) tool was employed to assess the strength of evidence and determine its generalizability. Electronic nicotine delivery systems (ENDS) have diverse mechanisms of toxicity, including inflammation, hypoxia, cardiovascular stress, and metabolic changes. Reported adverse effects include cough, throat irritation, nausea, and hemodynamic changes. However, ENDS are associated with fewer risks compared to conventional cigarette smoking. ENDS users experience fewer respiratory and cardiovascular issues and have lower levels of biomarkers such as NNAL and CO compared to traditional smokers. Additionally, ENDS are more effective than nicotine replacement therapy (nicotine patches) for smoking cessation, particularly in pregnant women. The side effects of ENDS and nicotine-free vaping are similar to those of conventional smoking in pregnant women, with the exception of a lower birth weight among newborns exposed to ENDS (p < 0.05). ENDS present a complex balance of benefits and risks regarding respiratory health. While there are adverse effects, ENDS are considered less detrimental than conventional smoking and a viable option for smoking cessation. Longitudinal studies are needed to evaluate their safety with long-term use (>16 weeks). Policymakers and health practitioners should use these findings to develop balanced public health policies that weigh the benefits of ENDS against potential health risks, enabling informed decision-making for users.

## Introduction and background

The rise in the use of electronic nicotine delivery systems (ENDS), commonly called vaping, has drawn concern on the effects on human health, most especially on the respiratory system [1]. People around the world have diverse views or attitudes toward vaping or ENDS. The use of ENDS has been growing rapidly and is present across the globe, with research suggesting that 82 million people vape in 2021 [2]. Although e-cigarettes are believed by some to be beneficial to tobacco smoking cessation, there have been worries about how helpful it is to the population in general and the youths in particular [3]. However, available facts suggest that ENDS are not harmless devices; they bear known threats and dangers of exposing their users to toxicants, nicotine, and carcinogens, perpetuating the risks of both lung diseases and injury to lung connective tissues similar to smokers [4,5]. Although these devices are aimed at being a much safer alternative to traditional cigarettes, the findings of recent studies indicate that vaping may be highly detrimental to respiratory health [6]. ENDS use has been associated with an increased probability of initiation of cigarette smoking among adolescents and less probability of smoking cessation, further highlighting the need for an extensive prevention strategy and policy aimed at dissuading susceptible population groups from establishing a pattern of tobacco and electronic cigarette consumption [1,4].

Therefore, in order to conduct a systematic evaluation of the toxicity of vaping on the respiratory system, it is mandatory to propose an extensive theoretical model that combines multiple pathways and determinant factors that vaping might have an impact on [7]. With reference to this model, it will be possible to define further the paths of harm and to determine the biomarkers of exposure that are essential for risk assessment, as well as the additional adverse effects resulting from short-term or long-term exposure. The key aspects of the review are the following.

Vaping aerosols that consist of chemicals, metals, and particles are inhaled and affect the respiratory system with various toxicities. The aerosols from ENDS have also been demonstrated to cause oxidative stress and irritation, DNA damage, and toxicity toward the individual’s cells, which suppresses their viability and formation of DNA adducts [8-10]. These aerosols contain toxic metals such as chromium, copper, and lead, cause elevation of reactive oxygen species (ROS) levels, reduction of glutathione, and affect cell viability, leading to DNA damage [8]. Moreover, the existence of nicotine, flavoring agents, and heating devices, all contained in e-vaping fluids, may also cause different unfavorable health effects that can affect the cardiopulmonary system, increase oxidative stress, and even cause genotoxicity [11]. These substances in the deposition within the surface of the respiratory system cause inflammatory effects and oxidative stress that lead to the development of a microenvironment supporting cancer or have a carcinogenic effect [12].

The proliferation of inflammatory cytokines such as TNF-α and IL-1β is observed after subjecting the individuals to vaping aerosols [13]. These events can result in the creation of ROS, which is a cause of oxidative stress and thus oxidative damage in the lung cells [12]. In addition, the direct toxigenic impact of vaping aerosols can elicit cell death and injury, impacting epithelial cells, macrophages, and other lung cells, leading to acute and chronic respiratory disorders [14]. To assess the potential respiratory disease risks from vaping and to design interventions in response, identifying and distinguishing the forms of inflammation, oxidative stress, and cellular damage resulting from vaping aerosols is fundamental.

Despite having been promoted as being safer than traditional cigarettes since they do not burn tobacco and produce smoke, vaping has been associated with detrimental respiratory health effects, including chronic obstructive pulmonary disease and other chronic lung diseases [15]. One notable and well-documented danger was the 2019 e-cigarette or vaping use-associated lung injury (EVALI) outbreak, which resulted in over 2,800 cases of severe lung injuries linked to vaping. This represents a significant acute risk associated with e-cigarette use [16]. While vaping is considered safe smoking due to the harm it causes by smoking, the research indicates that vaping is not harmless and is associated with cardiovascular disease, respiratory disorders, and the potential for addiction. The study also raises questions about the potential carcinogenic risks of vaping and the development of lung cancer in the long term [17]. Therefore, there is a need to conduct a study to determine the causes of lung injury due to vaping constituents to determine its effects on respiratory health [18].

There are similar negative repercussions attributable to conventional smoking and vaping, both of which are associated with the deterioration of oral health and well-being. While brushing the teeth, the use of fluoride has been found to enhance the chances of developing dental caries and periodontal diseases [19]. The harm caused by vaping involves inhaling an aerosolized liquid, which has concerning effects with changes observed for the oral cavity and discomfort [20]. Although vaping has been viewed as a helpful aid that helps quit smoking, it has been linked with conditions such as lung injuries like EVALI, which may be a result of substances such as vitamin E acetate interfering with the morphology and activity of lungs and lung surfactants [21]. Moreover, the use of addictive substances in networked e-cigarettes and alcoholic beverages can cause the consumption of other addictive substances; therefore, the use of e-cigarettes containing nicotine may result in the consumption of many other substances, such as alcohol and illicit drugs, owing to the gateway effect of nicotine [22]. Additionally, from a carcinogenicity point of view, exposure to both CS and e-cigarette aerosols elevated proinflammatory cytokine production and affected protein profiles, which raised lung cancer risk [23].

This theoretical framework provides a roadmap for examining the complex effects of vaping on the respiratory system in a more organized and thoughtful manner. Through systematically studying each aspect, beginning with inhalation and ending with chronic health effects, all of the toxic processes can be explained and helped to prevent at the population level and in the framework of regulating guidelines and policies. Finally, this model will help the systematic review to assess any existing gaps and emerging trends in order to make a suitable recommendation.

Owing to the rapid rise in the use of vaping, there is a perception that vaping is not as harmful as traditional smoking for respiratory system health. Moreover, some people are also encouraging the use of vapes because they consider that they are helpful in reducing the use of traditional smoking, which eventually helps smokers to stop smoking gradually. Therefore, the aim of this review is to examine the mechanism of toxicity and adverse effects on the respiratory system of individuals on the use of vapes while comparing the effects of the use of conventional smoking.

## Review

Methods

A systematic review was conducted using the Preferred Reporting Items for Systematic reviews and Meta-Analyses (PRISMA) guidelines [24]. PubMed, Embase, and Cochrane Library were searched using the following limiters: studies in the English language, studies from 2014 to 2024, open-access studies, peer-reviewed articles, and studies with full-text availability. To retrieve relevant literature, the concepts, text words, and Mesh words that were used to search relevant studies are mentioned in Table 1.

**Table 1:** PICO framework PICO, population/problem, intervention/exposure, comparison, and outcomes

PICO	Concepts	Text words	Controlled vocabulary
Population/problem	Adults who suffer respiratory or pulmonary injury due to vaping	Respiratory injury, pulmonary injury, pulmonary damage, e-cigarette or vaping use-associated lung injury	"Lung Injury"[Mesh]
Intervention/exposure	Vaping and electronic nicotine delivery systems	Vaping, electronic nicotine delivery systems, and e-cigarettes	"Vaping"[Mesh], "Electronic Nicotine Delivery Systems"[Mesh]
Comparison	None/conventional cigarettes	Cigarettes smoking	"Cigarette Smoking"[Mesh]
Outcomes	Lung functioning, toxicity, and adverse effects	Lung functioning, inflammatory markers, toxicity, and adverse effects	"Respiratory Function Tests"[Mesh], "Toxicity Tests, Chronic"[Mesh], "Long Term Adverse Effects"[Mesh]

Research Question

What is the mechanism of toxicity and adverse effects of ENDS on the respiratory system used among adults? How do ENDS differ in terms of effects on the respiratory system as compared to conventional smoking? Does ENDS use to encourage individuals to stop using cigarettes?

Inclusion Criteria

In this review, only randomized controlled trials (RCTs) in which adults of either gender used vaping or ENDS in routine either for the first time or were former users and quit just before less than six months were included. The studies from the last 10 years (2014-2024) were included. All studies in English, full text, peer-reviewed, and open-access published studies were considered. Only studies focused on the effects of vaping on the respiratory system or compared with conventional cigarettes were selected.

Exclusion Criteria

Observational studies, case reports, case series, retrospective case series, retrospective chart reviews, systematic reviews, narrative reviews, meta-analyses, letters of editors, and communications were not included. Studies before 2014 were excluded, not in English, paid, and published in non-peer-reviewed journals. Studies focused on topics other than vaping or nicotine delivery systems were not considered.

Studies Selection Process

The study selection process followed the PRISMA guidelines by two independent reviewers. Initially, 179 studies were identified, and 35 duplications were removed using Endnote X9, with the remaining 144 undergoing screening. While screening, abstracts, titles, and in-depth reading help exclude 58 irrelevant studies. The eligibility status of the remaining 86 studies was checked, and only 42 studies met the inclusion criteria, whose quality assessment was performed.

Quality Assessment

The two independent reviewers used Cochrane Risk of Bias 2.0 to assess the risk of bias, categorizing studies into high, low, and uncertain risks of bias [25]. The Grading of Recommendations Assessment, Development and Evaluation (GRADE) tool was used to determine the strength of the recommendation of trials, e.g., high quality for low ROB, moderate quality for uncertain ROB, and low quality for high ROB. Only high-quality studies were considered for this systematic review to ensure robustness and trustworthiness [26].

Data Extraction and Synthesis

Two independent reviewers extracted data from included studies, including study designs, sample size characteristics (age, gender, vaping, controls, adverse effects, and toxicity), vaping or ENDS, conventional smoking, comparator, outcomes measures, adverse effects, toxicity, objectives, contributions, and methodological quality assessment, and entered them in an Excel spreadsheet. The datasheet also contained information about conflict of interest among authors, data availability, ethical concerns, and the number of times the articles were cited.

The analysis of the studies was done using a systematic approach. A thematic analysis using an inductive, data-driven approach was considered [27]. It involves the in-depth analysis of the convergence of these results and a review through an iterative approach. The critical appraisal of the results of the theme was to analyze the evidence to ensure an informed, evidence-based understanding of novel clinical and safety outcomes for antiviral drugs.

Review

**Figure 1:**
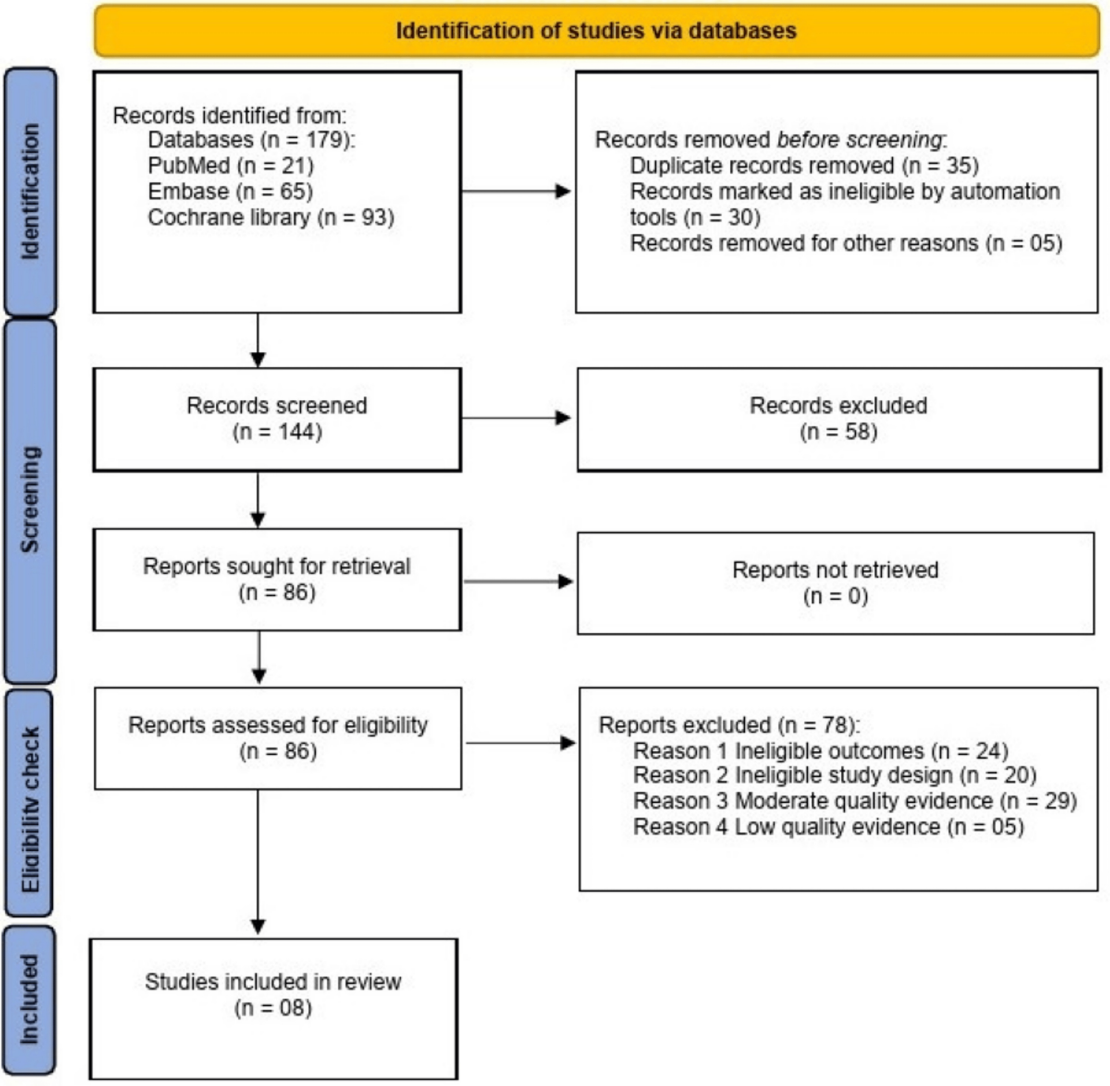
PRISMA flowchart Reason 1: irrelevant outcomes; reason 2: irrelevant study design; reason 3: moderate quality; reason 4: poor quality = 05. PRISMA, Preferred Reporting Items for Systematic Reviews and Meta-Analyses

The review was conducted following PRISMA guidelines to ensure the best evidence-based practice that reproduces results in the future by other authors (Figure 1).

This PRISMA-based review synthesized evidence. Initial searches of PubMed, Embase, and Cochrane library databases yielded 179 articles using keywords, text words, and controlled vocabulary. The 35 duplicate articles were removed by the EndNote x9 duplication finding tool. After duplication, the 144 articles were selected for screening. While screening, 58 irrelevant articles were excluded by reading titles and abstracts. Forty-two RCTs met inclusion criteria by reading in-depth research and focusing on selection criteria and review outcomes, which were methodologically assessed.

Assessment of Risk of Bias

The Cochrane Risk of Bias 2.0 tool evaluated the methodological quality of 42 RCTs across five domains. According to ROB 2.0, the trials were categorized into three groups: five trials were identified as high-risk, eight as low-risk, and 29 as having uncertain bias. The review ultimately included the eight low-risk RCTs.

GRADE Tool

The eight included RCTs had a low bias. The GRADE tool upgraded them to high quality while assessing the study based on six GRADE domains. Additionally, 29 RCTs with unclear bias risk downgraded evidence to “moderate quality.” The remaining five RCTs were reported with a high risk of bias and assigned as “low quality” (Table 2).

**Table 2:** Methodological quality assessment GRADE, Grading of Recommendations Assessment, Development and Evaluation

Serial number	Author	Risk of bias	Evidence strength (GRADE)	Evidence commentary
1	Masiero et al. (2019) [28]	Low risk of bias	High quality	In this double-masked, randomized controlled trial, 657 smokers were enrolled in the study. The research has been cited 51 times.
2	Franzen et al. (2018) [29]	Low risk of bias	High quality	This study is a single-center, double-blinded, randomized controlled trial involving 15 active smokers. It includes a control group and is registered. The research acknowledges its limitations and has been cited 125 times.
3	Hajek et al. (2019) [30]	Low risk of bias	High quality	In this unblinded, randomized study conducted across four centers, 886 smokers were enrolled. The research is registered, acknowledges its limitations, and provides comprehensive data. It has been cited 45 times.
4	Pulvers et al. (2020) [31]	Low risk of bias	High quality	This unblinded, randomized study with a control group included 186 participants and demonstrated a large effect size. It is registered, acknowledges its limitations, and has been cited 65 times.
5	Chaumont et al. (2020) [32]	Low risk of bias	High quality	This unblinded, randomized study involved 21 participants and reported a large effect size. It is registered, acknowledges its limitations, provides data, and has been cited 21 times.
6	Klonizakis et al. (2022) [33]	Low risk of bias	High quality	This single-center, randomized study included 248 participants and reported a large effect size. It acknowledges its limitations, provides data, and has been cited eight times.
7	Hajek et al. (2022) [34]	Low risk of bias	High quality	This randomized study, which included 1,140 participants and reported a medium effect size, is registered, acknowledges its limitations, and provides comprehensive data. It has been cited 29 times.
8	Przulj et al. (2023) [35]	Low risk of bias	High quality	This multi-center, randomized, placebo-controlled study involved 1,140 pregnant daily smokers, reported a medium effect size, is registered, acknowledges its limitations, and provides comprehensive data.

Characteristics of Included Studies

The characteristics of the included eight RCTs, categorized as “high quality,” included 3,855 patients, with the majority of patients being adults and more than 65% female. E-cigarettes were tested among former smokers to determine their safety and whether they had a lower adverse effect on ENDS than conventional smoking and nicotine replacement therapy (NRT). The effect of an ENDS was compared to equal and comparable controls to determine the effectiveness, which is shown in Table 3, along with adverse events, to ensure that ENDS are relatively safer than other smoking methods. Table 4 presents the key highlights and findings of the included studies.

**Table 3:** Characteristics of the included studies %FMD, percentage of flow-mediated dilation; ACh, acetylcholine; CO, carbon monoxide; CVC, cutaneous vascular conductance; DBP, diastolic blood pressure; ENDS, electronic nicotine delivery systems; FTCD, Fagerström Test for Cigarette Dependence; HAD, Hospital Anxiety and Depression Scale; LCQ, Leicester Cough Questionnaire; LFT, liver function tests; NRT, nicotine replacement therapy; NNAL, 4-(methylnitrosamino)-1-(3-pyridyl)-1-butanol; SBP, systolic blood pressure; SF-IPAQ: Short Form International Physical Activity Questionnaire; SNP, sodium nitroprusside

Author	Country of study	Sample size characteristics	Exposure	Control	Duration	Mechanism of toxicity	Biomarkers	Adverse effects	Outcomes	Limitations	Conclusion
Masiero et al. (2019) [28]	Italy	210, 132/78, 62.80 + 4.597 years	ENDS	CS and placebo	12 weeks	-	Peripheral and central hemodynamics and arterial stiffness parameters	Burning throat, cough, nausea, headache, insomnia, stomachache, confusion, and dyspnea	Level of CO, respiratory system, LCQ, Fagerstrom Test for Nicotine Dependence, and HAD	Higher drop rates in the control group, motivation participation with more desire to use e-cigarettes than quitting, missing data, lack of a systematic quantitative assessment of e-cigarette data, and measurement bias	All participants reported a significant reduction of tobacco consumption compared to the baseline; the use of e-cigarettes allowed smokers to achieve a better result (p < 0.05*)
Franzen et al. (2018) [29]	Germany	15, 5/10, 22.9 ± 3.50 years	ENDS	Nicotine-free vaping and CS	Four months	-	Peripheral and central hemodynamics and arterial stiffness parameters	Increased cardiovascular risk	HR, SBP, and DBP	Small sample size, intensity cannot be standardized, variance in e-cigarette composition (solvents), and high concentration of nicotine	Peripheral SBP increased significantly for 45 minutes after vaping nicotine-containing liquid (p < 0.05) and 15 minutes after smoking a conventional cigarette (p < 0.01), while nicotine-free liquids did not significantly change blood pressure during the first hour of follow-up
Hajek et al. (2019) [30]	UK	886, adults, >18 years	ENDS	NRT	12 weeks	-	-	E-cigarette users had significantly less coughing and phlegm at one year than NRT users	CO-validated sustained abstinence rates at 52 weeks, reduction in smoke intake, treatment adherence, and ratings, elicited adverse reactions, and changes in self-reported respiratory health	Results may not apply to other smokers, settings, or cartridge-based e-cigarettes	ENDS is more effective than NRT (p < 0.001*)
Pulvers et al. (2020) [31]	USA	186, 111/75, 43.3+12.5 years	ENDS	CS	Six weeks	-	-	Greater reduction in respiratory symptoms in the ENDS group (RR, 0.63 (95% CI, 0.47-0.85); p = 0.002)	Reduction in urinary NNAL concentration at week 6; secondary outcomes were changes in urinary cotinine, expired CO, respiratory symptoms, lung function, and blood pressure	One participant missed data for NNAL variables, cross-over trial, study period of six weeks was insufficient to estimate the effect of ENDS; the results were only limited to NSPS e-cigarettes	E-cigarettes may help African American and Latinx smokers reduce harm. (p < 0.001*)
Chaumont et al. (2020) [32]	Belgium	30 male participants with a mean age of 38.0 ± 2.0 years	ENDS	Nicotine-free vaping	Five days	E-cigarettes use propylene glycol and glycerol to vaporize liquid and transport nicotine. These small hydrophilic molecules quickly cross the lung epithelium. A trial hypothesized that regular vapers could completely clear aerosol deposits from the lungs and reverse cardiorespiratory toxicity by short-term cessation of vaping	Biological/clinical cardiorespiratory parameters, LFTs, serum nicotine, serum and urine propylene glycol, and serum CC16	Short-term vaping cessation altered urine metabolome and increased serum club cell protein-16, reducing lung inflammation. Due to lung gas exchange disturbances, acute vaping with and without nicotine decreased transcutaneous oxygen tension slightly	Serum/urine pneumoproteins, hemodynamic parameters, lung-function test and diffusing capacities, transcutaneous gas tensions (primary outcome), and skin microcirculatory blood flow may be reversible	Although the cessation period was short but deviated toward a cardiorespiratory healthy profile, small sample size, did not monitor the vaping condition in five days, results were dependent upon make population only, participants were former tobacco smokers their SpO2 may be lower relative to age	Higher club cell-16 protein, heart rate, and skin oxygen tension reduction was more in nicotine vaping (p < 0.05*)
Klonizakis et al. (2022) [33]	UK	248, 124/124, 44.0+13.0 years	ENDS	Nicotine-free vaping and NRT	Three to six months	-	Macrovascular function by %FMD, CVC responses to ACh and SNP as indicators measured using laser doppler fluximetry and Iontophoresis to assess upper body microvascular	-	Carbon monoxide, body mass index, blood pressure, number of cigarettes and years smoked, and physical activity measured using the SF-IPAQ	A single device and manufacturer were used to ensure consistency and standardization, with no group of continuing smoking included; as smokers, all participants were de facto controls for themselves, and no vasculature improvements are expected over time	Quitting smoking improved cardiovascular health after three and six months. Neither nicotine-containing nor nicotine-free e-cigarettes or NRT had superior cardiovascular benefits
Hajek et al. (2022) [34]	UK	1,140 pregnant females, 26.6 (22.5-30.9)	ENDS	NRT	Four weeks	-	FTCD, saliva samples for assessment of their cotinine level; salivary cotinine (<10 ng ml^−1^), salivary anabasine (<1 ng ml^−1^), and carbon monoxide level <8 ppm	Miscarriage, neonate death, preterm birth, NICU admission, congenital abnormalities, and adverse birth outcomes were similar in both groups (p > 0.05) as compared to low birth weight which is less frequent in ENDS (p = 0.01)	Self-reported abstinence and safety outcomes	Small volume and timing issues, challenges in biochemical validation, the impact of the COVID-19 pandemic, reduced validated quit rates	E-cigarettes outperformed patches (6.8% vs. 3.6%; RR = 1.93, 95%CI: 1.14-3.26, p = 0.02)
Przulj et al. (2023) [35]	England and Scotland	1140 pregnant smokers	ENDS	NRT	Two to eight weeks	-	-	Respiratory symptoms were reported in ENDS vs NRT (84.4% vs 79.8%) at the start. The e-cigarette arm had fewer infants with low birthweight (<2,500 g) (9.6% vs. 14.8%, RR = 0.65, 95% CI = 0.47-0.90; Bayes factor = 10.3), despite similar adverse events and birth outcome rates	Validated prolonged abstinence at the end of pregnancy	Low validation rates weakened the study. Many participants did not use the support enough to test its benefits, and the small sample size may have prevented the detection of less common adverse effects	ENDS are more safer and effective than NRT. Validated sustained abstinence rates were low (6.8% vs. 4.4% in e-cigarettes and nicotine patches, risk ratio = 1.55, 95% CI 0.95-2.53; Bayes factor = 2.7)

**Table 4:** Key contribution findings of the included studies DBP, diastolic blood pressure; EC, e-cigarette; ENDS, electronic nicotine delivery systems; NRT, nicotine replacement therapy; NP, nicotine patches; PWV, pulse wave velocity; SBP, systolic blood pressure; TcPO2, transcutaneous oxygen pressure

Serial number	Author	Key findings
1	Masiero et al. (2019) [28]	This study found that e-cigarettes were more effective in helping chronic smokers with high awareness of smoking-related risks to quit and reduce their daily cigarette consumption compared to a control group. Personalized medicine could leverage e-cigarette protocols and innovative ICT-driven self-management models to support behavioral changes and manage side effects. While the participants were motivated to quit, less motivated smokers in clinical settings might also benefit from such approaches.
2	Franzen et al. (2018) [29]	This study investigated the effects of ENDS, both nicotine-free and nicotine-containing liquids, on blood pressure in 15 young, active smokers. It also assessed acute peripheral and central hemodynamics and PWV in a control group using nicotine-free e-cigarettes. The findings suggest that, similar to cigarettes, nicotine-containing devices may elevate cardiovascular risk due to their higher parameters. Further research is needed to explore the chronic effects of both nicotine-containing and nicotine-free e-liquids on blood pressure.
3	Hajek et al. (2019) [30]	This study found that e-cigarettes are both more effective and cost-effective than NRT for smoking cessation. E-cigarettes demonstrated superior performance compared to NRT in multisession treatments for smokers seeking assistance. SSSs could enhance success rates, cost-efficiency, and the appeal of smoking cessation programs by providing e-cigarette starter packs.
4	Pulvers et al. (2020) [31]	This randomized clinical trial found that adult smokers who switched to a nicotine salt pod system had lower brief NNAL levels compared to those who continued smoking. ENDS may serve as an effective alternative for reducing smoking, particularly among African American and Latinx populations.
5	Chaumont et al. (2020) [32]	This randomized crossover study evaluated the baseline serum and urine metabolome, lung function, and cardiovascular parameters of regular e-cigarette users following short-term cessation. The findings revealed that daily ENDS users experienced a reduced heart rate, but increased levels of CC16 and FEF-25%, indicating improved airway status. Five days of vaping cessation altered urine metabolomics. Acute nicotine- and nicotine-free vaping led to transient lung gas exchange disturbances, resulting in decreased transcutaneous oxygen tension (TcpO2). Nicotine vaping specifically raised SBP, DBP, and heart rate acutely.
6	Klonizakis et al. (2022) [33]	This study compared the effects of nicotine-containing e-cigarettes, nicotine-free e-cigarettes, and NRT on smokers’ cardiovascular risk factors and health-related quality of life. The results showed cardiovascular benefits for smokers who quit, observed at both three and six months. However, neither nicotine-containing e-cigarettes, nicotine-free e-cigarettes, nor NRT demonstrated superior cardiovascular benefits.
7	Hajek et al. (2022) [34]	E-cigarettes were found to be more effective at promoting abstinence and had similar safety outcomes compared to NRT for smoking cessation in pregnant women. However, the unadjusted primary analysis did not conclusively demonstrate that e-cigarettes were superior to NRT in helping pregnant women quit smoking. The use of e-cigarettes within the NRT group may have obscured their effects. When excluding users of non-allocated products, e-cigarettes outperformed patches in all abstinence outcomes. For pregnant women who are unable to quit smoking, e-cigarettes appear to be no more dangerous than nicotine patches and may help reduce the risk of low birth weight.
8	Przulj et al. (2023) [35]	It was found that ECs were more effective than NPs in helping pregnant smokers quit and in reducing the likelihood of low birth weight. Overall, e-cigarettes represent a viable alternative tool for promoting smoking cessation among pregnant women. This is particularly significant as there are few effective interventions available for this important group, and e-cigarettes can aid pregnant women in transitioning away from traditional smoking.

ENDS Mechanism of Toxicity

Chaumont et al. (2020) investigated various mechanisms of vaping toxicity, including aerosol constituents, health effects, serum and metabolomic changes, and metabolic impacts [32]. E-cigarette liquids (e-liquids) consist of propylene glycol and glycerol, and when a user vapes, they inhale the aerosol produced by heating these liquids, which contain both flavorings and nicotine. High-wattage vaping, whether with or without nicotine, has been linked to transcutaneous hypoxia, airway constriction, and lung inflammation. Long-term exposure to these aerosols may elevate serum levels of club cell secretory protein-16 (CC16), which is associated with inflammation and potential lung damage.

At baseline, serum CC16 levels were lower during cessation, suggesting possible lung recovery or reduced injury if vaping is discontinued. Pulmonary nicotine vaping was associated with acute increases in systolic blood pressure (SBP), diastolic blood pressure (DBP), and heart rate, indicating cardiovascular stress. A five-day cessation from vaping led to changes in the metabolomic profile of urine samples, potentially reflecting alterations in metabolism related to toxin elimination or homeostatic adjustments. Acute changes were also observed in pulmonary gas exchange, with both nicotine- and nicotine-free vaping showing reduced transcutaneous oxygen tension (TcpO2).

Critically, the study highlighted the impact of vaping on respiratory parameters, noting significant positive correlations between changes in oxygen saturation (SpO2) and TcpO2 following both acute nicotine and nicotine-free vaping. Overall, these findings underscore the diverse toxicological effects and potential adverse health impacts of vaping, including inflammation, hypoxia, cardiovascular pressure, and metabolic alterations. The data on serum and urine biomarkers, along with respiratory and cardiovascular changes, suggest that vaping has biological effects and can be toxic.

Adverse Effects of ENDS

The reviewed eight RCTs provide a comprehensive overview of the impact of ENDS on respiratory health in both younger and adult populations. Masiero et al. (2019) found that participants using ENDS experienced a range of adverse effects, including burning throat, cough, nausea, headache, insomnia, stomachache, confusion, and dyspnea. Despite these side effects, there was a significant reduction in tobacco use compared to baseline (p < 0.05) [28]. Franzen et al. (2018) reported that nicotine-containing ENDS led to a significant increase in peripheral SBP and heart rate, indicating cardiovascular stress, which impacts respiratory health due to the interconnected nature of cardiovascular and respiratory systems (p = 0.01) [29]. Hajek et al. (2019) observed that ENDS users had a significantly reduced incidence of coughing and phlegm at one year compared to those using NRT, with improved respiratory symptoms overall (p = 0.001) [30]. Pulvers et al. (2020) found a significant decrease in respiratory symptoms among ENDS users and noted a reduction in urinary NNAL, a tobacco-specific nitrosamine biomarker (p = 0.001) [31]. Chaumont et al. (2020) showed that temporary cessation of vaping altered urine metabolome composition and increased serum levels of CC16, associated with lung inflammation, suggesting reversible detrimental effects on the lungs (p < 0.05) [32]. Klonizakis et al. (2022) found no superior cardiovascular benefits from nicotine-containing or nicotine-free e-cigarettes compared to NRT, although respiratory outcomes were not reported (p < 0.0001) [33]. Hajek et al. (2022) found that ENDS led to a lower incidence of low birth weight among pregnant women compared to NRT, although respiratory side effects were not a primary focus (p = 0.02) [34]. Przulj et al. (2023) reported that ENDS had a low association with issues such as low birth weight and respiratory symptoms, suggesting a potential reduction in harm (p = 0.04) [35].

Implications of the Respiratory System on the Use of ENDS and Cigarette Smoking

The implications of the respiratory system by making comparisons of ENDS with traditional smoking were determined in three RCTs [29-31]. ENDS seem to cause less detrimental effects on the cardiorespiratory system and are less carcinogenic as compared to conventional smoking. Franzen et al. (2018) reported that during the first hour of observation, vaping nicotine-containing liquid resulted in a significant increase in peripheral SBP for 45 minutes (p < 0.05), while smoking a conventional cigarette led to a significant increase for 15 minutes (p < 0.01). However, nicotine-free liquids do not affect blood pressure. The additional statistics revealed a notable rise of more than 5% in DBP in the Cig arm (p < 0.05). In contrast to these results, there was a significant reduction of greater than 4% in DBP after an interval of 30 minutes (p < 0.05). The act of smoking cigarettes resulted in a heart rate increase of more than 8% within the initial 30 minutes, with statistical significance (p < 0.05). Further data indicates a notable reduction in DBP within 30 minutes of smoking or vaping (p < 0.01 and p = 0.005), while there is a tendency for an increase in the conventional cigarettes group within 15 minutes (p = 0.064). However, the results are limited to generalization because of the small sample size, variance in composition of e-cigarettes, and high concentration of nicotine in e-cigarettes [29].

Hajek et al. (2019) also found that in ENDS users, there were noted fewer incidences of symptoms like cough and phlegm as compared to the adults using conventional smoking [30]. However, Masiero et al. (2019) still reported side effects such as burning throat and dyspnea in ENDS users [28]. Pulvers et al. (2020) found that compared to baseline, e-cigarette users had lower rates of NNAL, CO, cigarette consumption in the past week among smokers, and respiratory symptoms at week 6 than conventional smokers (CSs). Furthermore, e-cigarettes reduce carcinogenicity. To conclude, ENDS may be used as an alternative or reduction strategy tool among African Americans and Latinx (p < 0.05). The evidence synthesizes that e-cigarettes are reported to be less harmful as compared to conventional smoking due to their lower levels of carcinogens, potentially lesser cardiorespiratory risks, and fewer changes in the hemodynamics of users.

Role of ENDS for Cessation of Cigarette Smoking

The role of ENDS in the cessation of conventional cigarette smoking has been discussed in three RCTs. To determine the e-cigarettes efficacy in smoking cessation, e-cigarettes and more so nicotine salt pods have better cessation rates as compared to nicotine patches and conventional cigarettes. To a greater extent, e-cigarettes are less detrimental or a bit safer as per biomarkers, e.g. NNAL, CO, and respiratory symptoms in African Americans and Latinx smokers [31]. Moreover, Hajek et al. (2022) and Przulj et al. (2023) studied the role of e-cigarettes among pregnant women to stop conventional smoking to reduce the detrimental effects of conventional smoking. Hajek et al. (2022) found that e-cigarettes outperformed nicotine patches and helped to reduce smoking cessation among pregnant women with a reported relative risk ratio of 1.93 at 95% CI (p = 0.02) [34]. Similarly, Przulj et al. (2023) also found that ENDS use is safer and more effective than NRT with a relative risk ratio of 1.55 at 95% CI to validate the abstinence rate in ENDS and nicotine patches are relatively similar. However, the adverse events were similar in e-cigarettes, NRT, and CSs except for low birthweight, which is less reported in e-cigarette users [35]. In the synthesis of evidence, e-cigarettes seem to be a viable option for smoking cessation and reduction measures; however, there are cardiovascular and respiratory issues that warrant more attention.

Discussion

The systematic review synthesized evidence that the mechanism of ENDS toxicity can present in diverse ways, such as inflammation, hypoxia, cardiovascular stress, and metabolic changes. ENDS reported adverse events (e.g., burning throat, cough, nausea, and hemodynamic changes), whereas ENDS also reduced cardiorespiratory parameters compared to conventional smoking. The research has shown that ENDS have fewer respiratory and cardiovascular implications than conventional cigarette smoking, with transient increases in hemodynamic parameters such as blood pressure and pulse rate among ENDS users compared to prolonged changes among CSs. Further, ENDS have demonstrated beneficial effects for smoking cessation, particularly for pregnant women that did significantly better than NRT (nicotine patches) and resulted in fewer low birthweight. In conclusion, ENDS can be regarded as a viable strategy to minimize harm.

The mechanism of electronic nicotine toxicity involved inflammation, hypoxia, cardiovascular stress, and metabolic changes reported in this review. Nicotine is the possible culprit to cause toxicity used in ENDS. Vieira-Alves et al. (2020) reported that nicotine, a specific cholinergic agonist, speeds up the development of atherosclerosis (AS) by activating nicotinic acetylcholine receptors found in both neural and non-neural tissues [36]. However, these findings are aligned with the study of Fu et al. (2021), who demonstrated that nicotine promotes AS by stimulating nicotinic acetylcholine receptors. It regulates the dysfunction of endothelial cells, vascular smooth muscle cells, and immune cells that contribute to the onset and progression of AS. Moreover, it activates growth factors and ROS, leading to abnormal lipid metabolism and inflammation in AS [37]. Therefore, the findings suggest that nicotine-free ENDS must be encouraged among e-cigarette users to avoid toxicity caused by nicotine. It is also suggested that the design and manufacturing of ENDS should take this evidence into account to mitigate harmful inflammatory responses, cardiovascular stress, and metabolic changes. It can be done through future researchers to conduct trials of nicotine-free for long-term health effects, or in another case, lowering the intensity of nicotine because this review is unable to consider any trial that focused on various intensities of nicotine to determine health implications.

ENDS caused adverse effects such as burning throat, cough, nausea, and changes in hemodynamics, as reported in this review. However, the use of ENDS among CSs is reported to be less detrimental because it is considered less harmful as compared to the well-established harmful CS constituents. Similarly, Ashour (2023), while reviewing 87 clinical trials to determine the adverse effects of ENDS and the role of ENDS in smoking cessation, found that ENDS are less detrimental than combustible cigarettes. It was also observed in these four-week to 12-month clinical trials that ENDS not only helps CSs to effectively quit smoking but also indicates them as an alternative tool to use instead of conventional smoking because of its less detrimental effects. Existing evidence strongly suggests that electronic cigarettes are a considerably less detrimental option compared to smoking, and smokers who transition from tobacco to electronic cigarettes are anticipated to experience notable improvements in their health. Therefore, it is emphasized that the effectiveness of electronic cigarettes as alternatives to tobacco smoking enhances and provides clearer guidelines to minimize any remaining risks associated with their use. This can be achieved by implementing proper quality control measures and establishing appropriate standards [38].

E-cigarettes are beneficial for conventional smoking cessation, but particularly for pregnant women, which did significantly better than NRT (nicotine patches). The review directs focus on the role of e-cigarette smoking, which is found to be significant (p < 0.05). The review compared different smoking methods, such as e-cigarettes that are nicotine-containing, nicotine-free e-cigarettes, conventional smoking, and NRT (nicotine patches). The e-cigarettes were found to be more beneficial in smoking cessation in this review as compared to NRT. However, Worku and Worku (2019) indicate that e-cigarettes can have harmful effects on various cell lines and animal models due to their flavorings and nicotine content. However, these effects have not resulted in significant health consequences after a follow-up period of 3.5 years. Nevertheless, e-cigarette use has been associated with the development of chronic lung disease and cardiovascular disease. Although marketed as a reliable method for quitting smoking, there is no agreement on their effectiveness, despite the initial strong evidence from a well-designed RCT that shows some positive outcomes. However, this is counterbalanced by the fact that the most prevalent use of e-cigarettes is as a dual user, and there is evidence indicating a threefold higher risk of future tobacco smoking [39]. The argument and counterargument emphasize the future need to do longitudinal trials to specifically focus on resolving this debate to standardize the guidelines in public health programs.

The review also found that among pregnant women, e-cigarettes showed a better strategy to stop smoking tobacco cigarettes and were also significantly better than NRT. The adverse events reported to be similar among these three smoking methods except for the low birth weight of newly born infants in e-cigarette users. Therefore, smoking cessation and low birth weight were two positives that encouraged e-cigarette use among pregnant women. In the United Kingdom, Lutman-White et al. (2024) also found that e-cigarettes indicated positive outcomes and effective strategies within smoking cessation services among pregnant women with the intention of quitting smoking [40]. Although there are some barriers to quitting smoking by women, which may further improve cessation support. It is not answered in the included studies, which direct future researchers to explore this aspect further.

Strengths and Limitations

The standardized tools were used to assess the methodological and risk of bias assessment of included studies to grade the evidence strength of recommendation. The evidence is synthesized by including only high-quality evidence studies. The ENDS mechanism of toxicity, adverse events, the relative safety of ENDS, and the role of ENDS in smoking cessation are determined, which may be incorporated into national or regional policy to curb conventional smoking and lead individuals to a healthy cardiorespiratory profile.

The review has reported the following limitations while synthesizing evidence from RCTs: a higher dropout rate from the control group and participants’ desire to vape more than to quit than the e-cigarettes’ effectiveness. Measurement bias was also a concern since there was little standardized quantitative evaluation of e-cigarette information and evidence. These limitations included a small sample size and an inability to control the strength/volume/duration of vaping and the specific e-cigarette formulation. Such findings cannot be extrapolated to other smokers, other contexts, or other forms of cartridge-based e-cigarette use. Cross-sectional, like crossover design, and a short study duration of six weeks were unable to capture the impact of ENDS in their entirety. The study protocol was tested with only one device and of the same make; there was no control group for the participants who were asked to smoke continuously. These limitations, including biochemical validation challenges, COVID-19 pandemic effects, and lower validated quit rates, pulled the study down. This research was also limited by the low validation rates and the insufficient use of support among the participants, which may have masked some of the adverse effects and their frequency, given the small size of the sample (Table 3).

Implications and Future Recommendations

The findings provided in this systematic review of the eight completed RCTs have important implications for public health policy. Thus, although ENDS appear to pose fewer direct respiratory and cardiovascular risks than conventional cigarette smoking, they are not without negative or adverse effects. Examining ENDS use, there are signs of reduced use of conventional cigarette smoking and lessening the biomarkers such as NNAL, urine metabolome, hemodynamics parameters, and CO, which could be useful in harm reduction. Besides, these trials suggest that ENDS have potential for smoking cessation, particularly for pregnant women, and may be more effective than the nicotine patch. Nevertheless, the differences in the composition of e-cigarettes, few participants, and short examination periods suggest the need for extended and longitudinal studies for the comprehensive assessment of ENDS’ safety and the development of recommendations for their application in smoking cessation interventions. These results should guide public health campaigns in weighing the benefits of using ENDS in reducing harms against the possible health risks and help the user make the right decisions.

## Conclusions

The systematic review concluded that ENDS have both positive and negative implications. The evidence suggests that ENDS, due to their less harmful and less toxic impact on respiratory health compared to traditional smoking, can be beneficial for smoking cessation and for transitioning from conventional smoking to ENDS use. While ENDS can cause inflammation, hypoxia, cardiovascular stress, and metabolic changes, they pose significantly fewer risks than conventional cigarette smoking. The reviewed RCTs indicate that ENDS users experience fewer immediate respiratory and cardiovascular adverse effects and have lower levels of biomarkers such as NNAL and CO. Additionally, ENDS are shown to be more effective than NRT, such as nicotine patches, for smoking cessation, particularly among pregnant women, resulting in a lower incidence of low birth weight in newborns. However, the studies have limitations, including small sample sizes, short research durations (12-16 weeks), and variability in ENDS composition, which restricts the generalization of results to pregnant women alone. Despite these limitations, the overall evidence is applicable to the general population. ENDS appear effective in reducing harm and promoting smoking cessation, but further longitudinal studies are necessary to evaluate their long-term safety beyond 16 weeks and to establish consistent protocols. Policymakers and health practitioners should consider these findings when crafting public health policies that balance the benefits of ENDS against the potential health risks of traditional smoking, ensuring informed decision-making for users.
